# UCHINANCHU AMERICAN OLDER ADULTS’ INFORMATION PREFERENCES AND BEHAVIORS IN END-OF-LIFE DECISION-MAKING

**DOI:** 10.1093/geroni/igac059.2527

**Published:** 2022-12-20

**Authors:** Kristina Shiroma, Bo Xie

**Affiliations:** University of Texas at Austin, Austin, Texas, United States; The University of Texas at Austin, Austin, Texas, United States

## Abstract

End-of-life (EOL) decisions are unique and sensitive, requiring nuanced health information. Careful examination of ethnic minority older adults’ information preferences and behaviors in EOL decision making from a cultural perspective is increasingly necessary as the national and global population grows older and more diverse. Little is known about the information preferences and behaviors of Asian American older adults in EOL decision-making, especially for underrepresented ethnic minorities such as Uchinanchu American older adults. This pilot study examined Uchinanchu American older adult’s information preferences and behaviors in EOL decision making. In November 2019, I interviewed 4 Uchinanchu American older adults (age range: 67-85 years; Mean: 76 years; SD: 8.04) recruited from personal contacts and through Uchinanchu cultural groups in Texas and Hawaii. The interviews took place in-person and via phone. Each interview lasted approximately 90 minutes. Interviews were transcribed verbatim and analyzed using inductive thematic analysis. I identified three themes from the data: (1) EOL decision-making information not coming from healthcare professionals; (2) Funeral planning easier to talk about than advance EOL medical decision making; and (3) Cultural traditions may exclude people (women; non-first born children) from EOL decision making. My findings suggest possible misalignments of EOL communication between medical professionals and Asian ethnic minority older adults. These findings may inform the development of culturally competent interventions to enhance EOL decision making.

